# Rectus sheath hematoma masquerading as acute pancreatitis: a case report

**DOI:** 10.1097/MS9.0000000000004630

**Published:** 2026-01-02

**Authors:** Aleena Aman, Ayesha Malik, Muddassir Khalid, Kanika Gupta

**Affiliations:** a Department of Emergency Medicine, Nazareth Hospital, Philadelphia, PA, USA; b Department of Medicine, Nishtar Medical University, Multan, Pakistan

**Keywords:** Cullen’s sign, pancreatitis, rectus sheath hematoma

## Abstract

**Introduction and Importance:**

Rectus sheath hematoma (RSH) is an uncommon and frequently misdiagnosed cause of acute abdominal pain that may mimic more serious intra-abdominal emergencies, including acute pancreatitis.

**Case Presentation:**

We present the case of a 62-year-old man who developed a large RSH following a coughing episode, presenting with right-sided abdominal pain and periumbilical ecchymosis (Cullen’s sign). Normal pancreatic enzymes and computed tomography (CT) imaging were key in excluding pancreatitis and confirming the diagnosis of RSH. The patient was managed conservatively with a successful resolution.

**Clinical Discussion:**

This case highlights the importance of considering RSH in the differential diagnosis of acute abdomen, even in the absence of anticoagulation, and the critical role of imaging in its timely diagnosis. This case reinforces the value of CT imaging and focused physical examination in differentiating RSH from other causes of acute abdomen.

**Conclusion:**

RSH remains a rare but critical diagnostic consideration in patients presenting with acute abdominal pain, particularly when Cullen’s or Grey Turner’s signs are present but pancreatic enzymes are normal. Early diagnosis, facilitated by CT imaging, can prevent unnecessary surgical interventions and guide successful conservative management, thereby improving patient outcomes.

## Introduction

Rectus sheath hematoma (RSH) is a rare but important cause of abdominal pain, accounting for approximately 1–2% of cases presenting with acute abdomen^[1,2]^. It involves bleeding into the sheath of the rectus abdominis muscle, typically resulting from rupture of the superior or inferior epigastric arteries or tearing of muscle fibers^[3,4]^. Commonly associated risk factors include anticoagulation therapy, female sex, advanced age, recent abdominal surgery, and increased intra-abdominal pressure due to coughing, vomiting, or physical exertion^[5,6]^. While Cullen’s sign (periumbilical ecchymosis) and Grey Turner’s sign (flank ecchymosis) are classically associated with hemorrhagic pancreatitis, these signs can also appear in RSH, due to tracking of blood through fascial planes^[7]^. Consequently, RSH can masquerade as acute pancreatitis, potentially leading to diagnostic delays or unnecessary surgical interventions^[8,9]^. The condition is more often reported in older women, especially those receiving anticoagulation therapy^[6,10]^, but spontaneous cases in patients not receiving anticoagulants have been increasingly recognized^[4,11]^. Here, we report a case of spontaneous RSH in a male patient without anticoagulant use, whose symptoms mimicked acute pancreatitis. This case highlights the diagnostic importance of imaging and the clinical utility of physical exam maneuvers, such as Carnett’s sign, in distinguishing between abdominal wall and intra-abdominal pathologies. This case report has been reported in line with the SCARE checklist^[12]^.

**HIGHLIGHTS**

- Rectus sheath hematoma is a rare cause of acute abdominal pain.
- RSH can mimic intra-abdominal emergencies, including pancreatitis.
- Cullen’s sign with normal enzymes should prompt suspicion of RSH.
- CT imaging is crucial for accurate diagnosis and avoiding mismanagement.
- Conservative management can achieve successful outcomes in RSH.

## Case presentation

**Figure 1.**
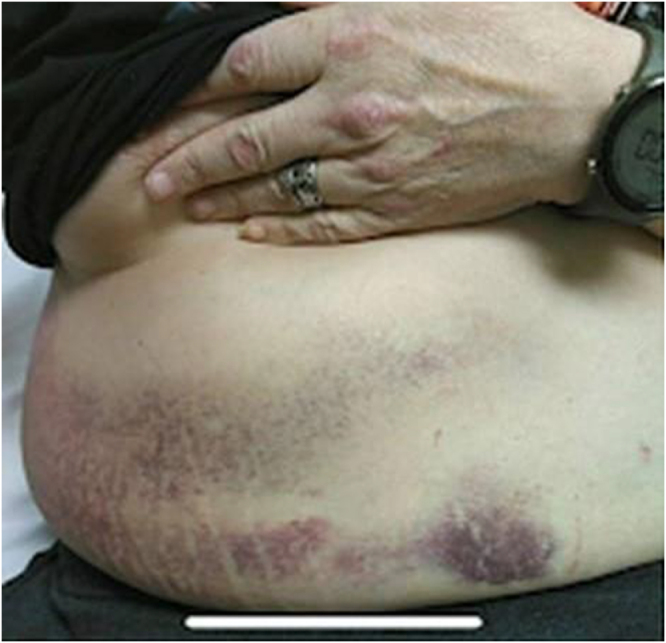
Cullen’s sign with a lower-quadrant mass.

A 62-year-old male with a history of type 2 diabetes mellitus, hypertension, moderate persistent asthma, and generalized anxiety disorder presented to the emergency department with sudden-onset right-sided abdominal pain and visible bruising. He reported the onset of symptoms following a prolonged and severe coughing spell during an asthma exacerbation. He reported that the pain had started suddenly about 24 hours before arrival, following a prolonged coughing episode. He denied trauma, recent abdominal surgery, or anticoagulant use. His only antiplatelet therapy was low-dose aspirin. On examination, the patient appeared anxious and had stable vital signs. Physical examination revealed faint periumbilical and right flank ecchymosis (Fig. 1). Palpation elicited marked tenderness and a firm mass over the right lower quadrant. Palpation elicited marked tenderness and revealed a firm, non-mobile mass in the right lower quadrant, measuring approximately 10–12 cm in length, corresponding to the subsequently identified RSH on computed tomography (CT). There were no peritoneal signs, and bowel sounds were normal.

### Investigations

**Figure 2.**
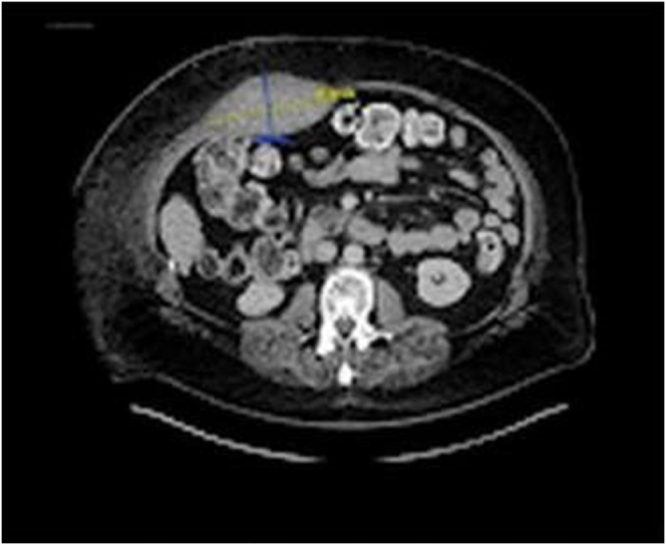
CT scan showing hematoma. CT, computed tomography

Initial laboratory tests revealed a mild decrease in hemoglobin (from baseline 14 g/dL to 11.8 g/dL) and normal serum amylase and lipase levels. Liver function and coagulation panels were unremarkable. Abdominal CT scan (Fig. 2) revealed a large hematoma within the right rectus abdominis sheath (12 × 6 × 3 cm), consistent with a Berna Type II RSH^[13]^. No signs of pancreatitis or intra-abdominal organ injury were present.

### Management

The patient was admitted for observation and managed conservatively with bed rest, low-dose opioid analgesia (tramadol 50–100 mg as needed) to avoid Non-Steroidal Anti-Inflammatory Drugs (NSAIDs) due to their bleeding risk, along with an abdominal binder and serial monitoring of vital signs and hemoglobin every 6 hours during the first 24 hours, followed by 12-hour intervals once he remained stable.

### Outcomes and follow-up

Over the next few days, his hemoglobin remained stable, ranging between 11.6 and 11.9 g/dL on serial measurements. His abdominal pain progressively improved, and he required no invasive intervention. He remained hospitalized for four days, during which he maintained hemodynamic stability and demonstrated adequate symptom control. He was discharged home with instructions to continue using the abdominal binder, avoid strenuous activity for two weeks, and attend follow-up in the surgical outpatient clinic. At his 2-week follow-up, he reported significant symptom resolution, with no recurrence or complications. The patient reported feeling reassured once the diagnosis was established and expressed significant relief with the gradual improvement of pain, noting that the abdominal binder and clear communication regarding the benign nature of the condition helped reduce his anxiety throughout hospitalization.

## Discussion

This case underscores the diagnostic complexity posed by RSH, particularly when it mimics more common intra-abdominal emergencies such as acute pancreatitis. While RSH is an uncommon etiology, accounting for only 1–2% of cases of acute abdominal pain, its clinical significance lies in its ability to masquerade as more serious conditions, leading to misdiagnosis, unnecessary interventions, and potentially worse outcomes^[1,2]^. In this patient, RSH was precipitated by a severe coughing episode – an established, though less frequently recognized, risk factor. Vigorous contractions of the abdominal wall, as occur during coughing, sneezing, or vomiting, can cause tearing of rectus muscle fibers or rupture of the epigastric vessels, particularly in elderly or frail patients^[3,4]^. Although anticoagulation is a predominant risk factor (reported in up to 70% of cases), spontaneous RSH can occur in patients not on anticoagulants, as seen in this case^[5,6]^. Low-dose aspirin may have contributed marginally, but the absence of therapeutic anticoagulation illustrates that RSH should not be excluded solely based on medication history.

The appearance of Cullen’s sign (periumbilical ecchymosis), as shown in Figure 1, traditionally associated with hemorrhagic pancreatitis or ruptured ectopic pregnancy, can also be seen in RSH due to tracking of blood through fascial planes to the subcutaneous tissue. This phenomenon, although rare in RSH, has been previously reported and was evident in our case within 24–48 hours after symptom onset, consistent with the literature^[7,8]^. The presence of Cullen’s sign in a patient with normal pancreatic enzyme levels should raise suspicion for alternative hemorrhagic sources, including RSH. Physical examination signs such as Carnett’s sign (persistence or worsening of tenderness with tensing of the abdominal wall) and Fothergill’s sign (a palpable mass that does not cross the midline and remains unchanged with muscle contraction) are critical yet often underutilized clinical clues to distinguish abdominal wall pathologies from intra-abdominal sources^[9,10]^. Imaging remains the cornerstone for accurate diagnosis of RSH. While ultrasonography can detect hematomas and is valuable at the bedside, it is operator-dependent and limited in obese individuals or when distinguishing intraperitoneal from abdominal wall pathologies^[11,14]^. CT, in contrast, is the gold standard for diagnosis with a near 100% sensitivity and specificity^[15]^. It provides detailed information on the location, extent, and potential active bleeding of the hematoma. In our patient, CT findings revealed a large right rectus sheath hematoma confined to the muscle, without signs of pancreatitis or intra-abdominal injury, aligning with a Berna Type II classification^[13]^.

Berna *et al*.’s classification stratifies RSH based on CT findings:

- Type I: small, confined intramuscular hematomas.
- Type II: moderate-sized hematomas between the muscle and transversalis fascia, without peritoneal involvement.
- Type III: large hematomas extending into the prevesical or intraperitoneal space, potentially causing hemodynamic instability^[13]^.

Management of RSH depends on the type of hematoma and the patient’s clinical status. Conservative management remains the mainstay for hemodynamically stable patients with Type I or II RSH, as well as for some stable Type III hematomas^[16]^. This includes bed rest, analgesia, ice or compression therapy, reversal or cessation of anticoagulation if applicable, and close monitoring of hemodynamics and serial hemoglobin levels^[17]^.

In this case, conservative therapy led to a favorable outcome without the need for invasive interventions. Literature supports this approach, with success rates exceeding 80% in retrospective series^[6,16]^. Indications for more aggressive treatment – such as arterial embolization or surgery – include ongoing bleeding, hemodynamic instability, or failed conservative therapy^[18]^. Angiographic embolization, particularly of the inferior epigastric artery, has been reported to be highly effective and is the preferred option over surgery, which carries significant morbidity and mortality risks, especially in older or anticoagulated patients^[19,20]^. The prognosis for RSH is generally favorable when diagnosed early and treated appropriately. Mortality remains low (<2%) in recent studies but can rise significantly with delayed recognition or in the presence of coagulopathy^[6,19]^. Complete hematoma resolution may take several weeks to months, depending on size and patient factors. Follow-up imaging may be warranted in large hematomas or if symptoms persist.

## Conclusion

This case highlights the importance of CT imaging and a focused physical examination in distinguishing RSH from other causes of acute abdominal pain. Early diagnosis can prevent unnecessary surgeries and guide effective conservative treatment. Increased clinician awareness is essential to reduce diagnostic errors and improve outcomes in patients with RSH This case highlights several key learning points: (1) RSH should be considered in the differential diagnosis of abdominal pain, especially in older adults with risk factors such as coughing, even in the absence of anticoagulation; (2) Cullen’s and Grey Turner’s signs are not pathognomonic for pancreatitis and may be misleading; (3) physical exam findings such as Carnett’s sign provide valuable diagnostic clues; and (4) early CT imaging facilitates accurate diagnosis, avoids unnecessary surgeries, and guides appropriate conservative versus interventional management. Increased awareness and a high index of suspicion for RSH are essential for timely diagnosis and optimal outcomes.

TITAN Guidelines: This manuscript complies with TITAN Guidelines, 2025, declaring no use of AI^[21]^.

## Data Availability

Data available on request from the authors.

## Peer and provenance statement

Not commissioned, externally peer-reviewed.

## References

[1] Alla VM Karnam SM Kaushik M. Spontaneous rectus sheath hematoma. West J Emerg Med 2010;11:76–79. 20411082 PMC2850860

[2] Costello J Wright J. Rectus sheath haematoma: a diagnostic dilemma? Emerg Med J 2005;22:523–24. 15983097 10.1136/emj.2004.015834 PMC1726840

[3] Cocco G Ricci V Amato B. Sonographic demonstration of a spontaneous rectus sheath hematoma following a sneeze: a case report. J Ultrasound 2021;24:125–30. 32621122 10.1007/s40477-020-00493-4 PMC8137746

[4] Iliklerden UH Kalayci T. Treatment of rectus sheath hematomas: eight years of single-center experience. Ulus Travma Acil Cerrahi Derg 2021;27:222–30. 33630287 10.14744/tjtes.2020.22893

[5] Cherry WB Mueller PS. Rectus sheath hematoma: review of 126 cases at a single institution. Medicine (Baltimore) 2006;85:105–10. 16609349 10.1097/01.md.0000216818.13067.5a

[6] Hatjipetrou A Anyfantakis D Kastanakis M. Rectus sheath hematoma: a review of the literature. Int J Surg 2015;13:267–71. 25529279 10.1016/j.ijsu.2014.12.015

[7] Devkota S Bista A Singh S. Spontaneous rectus sheath hematoma as a differential diagnosis for localized abdominal pain. Clin Case Rep 2024;12:e9436. 39434765 10.1002/ccr3.9474 PMC11491412

[8] Angeramo CA Faccini C Schlottmann F. Rectus sheath hematoma: conservative, endovascular or surgical treatment? Eur J Trauma Emerg Surg 2022;48:2157–64. 35031823 10.1007/s00068-021-01854-2

[9] Ural B Usta S Topuz O. Risk factors and management of rectus sheath hematoma. Ulus Travma Acil Cerrahi Derg 2016;22:441–45.27849320

[10] Mehta V Yadav S Yadav SS. Risk factors, classification, and clinical implications of rectus sheath hematoma. J Emerg Med 2019;57:292–97.

[11] Osman Y Abd El Maksoud W El Nakeeb A. The diagnostic role of ultrasonography in rectus sheath hematoma. Ultrasound Med Biol 2017;43:1212–17.

[12] Kerwan A Al-Jabir A Mathew G. Revised surgical CAse REport (SCARE) guideline: an update for the age of Artificial Intelligence. Prem J Sci 2025;10:100079.

[13] Berna JD Garcia-Medina V Guirao J. Rectus sheath hematoma: diagnostic classification by CT. Abdom Imaging 1996;21:62–64. 8672975 10.1007/s002619900011

[14] Chou JW Lin SH Lin CC. Diagnostic pitfalls in abdominal ultrasonography: rectus sheath hematoma mimicking intraperitoneal pathology. Am J Emerg Med 2014;32:1156.e5–1156.e7.

[15] Kang EJ Jeon SR Park Y. CT features and clinical characteristics of rectus sheath hematomas: a 10-year retrospective study. Abdom Radiol (NY) 2020;45:1992–99.

[16] Özucelik DN Duman S Hacım NA. Management of rectus sheath hematoma: conservative approach versus interventional techniques. Ulus Travma Acil Cerrahi Derg 2018;24:111–17.

[17] Hoefflinghaus PT Kampmeier TG Klink CD. Spontaneous rectus sheath hematoma: factors associated with morbidity. Eur J Intern Med 2021;92:60–64.

[18] El Hussein M Tuma F Zayed H. Management of massive rectus sheath hematoma: a systematic review. Am Surg 2020;86:1694–703.

[19] Ryu KH Park YS Joo SH. Predictive factors for clinical outcome and treatment in rectus sheath hematoma: a single-center experience. World J Emerg Surg 2020;15:62.33228705

[20] Koyuncu S Demirbas S Turan H. Rectus sheath hematoma: an emerging cause of acute abdominal pain. J Emerg Med 2019;57:615–21.

[21] Agha R Mathew G Rashid R. Transparency In The reporting of Artificial Intelligence – the TITAN guideline. Prem J Sci 2025. doi:10.70389/PJS.100082.

